# Factors and consequences associated with a delay in the discharge process of patients from an adult critical care unit

**DOI:** 10.1186/cc9883

**Published:** 2011-03-11

**Authors:** J Mellinghoff, A Rhodes, M Grounds

**Affiliations:** 1 St George's Healthcare NHS Trust, London, UK

## Introduction

Adult intensive care beds are a scarce and expensive resource. Efficient utilisation of these beds necessitates safe and timely discharge of patients to the general ward. However, the discharge process is complex and often delayed. This study aimed to look at the processes and consequences that cause a delay in the discharge of patients from an adult ICU.

## Methods

This was a retrospective study of our data collection databases based in a 17-bed London teaching hospital ICU. We examined the process of patient discharge from ICU to the ward over a 3-year period.

## Results

The study period was from July 2007 until June 2010. There were 3,511 patient discharge episodes to hospital wards. A delay of over 4 hours occurred in 2,829 patient episodes (81%). The delays in discharge to the wards increased by over 100% for the year following a reduction of 28 beds in total intrahospital ward bed capacity [1]. There were over 42,000 hours (equal to 1,751 days) of delays in discharges for the patient episodes. Delays were caused by all stakeholders involved in the discharge process. The main reasons were insufficient ward bed availability (21%), delays in bed allocation (30%), delays in the completion of administrative tasks on the ICU (4%), delays in adequate preparation of ward beds (27%) for the arrival of the ICU patient, and delays that were attributable to intrahospital transport arrangements (5%). Overall, discharge delays to surgical wards were twice as likely compared with medical wards as they were also trying to deal with elective and emergency surgical admissions. Medical wards had fewer delays in transfer but were more likely to have longer delay times as a result of subsequent delays in discharging patients back to the community.

## Conclusions

Delays were multifactorial and accumulative in nature and dependent on the individual processes involved in the transfer of patients. Themes were related to organisational, individual, teamwork and patient factors.

## References

[B1] Department of Health Overnight Bed Count http://www.dh.gov.uk/en/Publicationsandstatistics/Statistics/Performancedataandstatistics/Beds/DH_083781

